# Supplementary material for: CD19 CAR T Cells Expressing IL-12 Eradicate Lymphoma in Fully Lymphoreplete Mice through Induction of Host Immunity
Source: Mol Ther Oncolytics. 2017 Dec 19;8:41–51. doi: 10.1016/j.omto.2017.12.003 (PMC5772011; doi:10.1016/j.omto.2017.12.003)

OMTO, Volume *8*

Supplemental Information

CD19 CAR T Cells Expressing IL-12 Eradicate Lymphoma in Fully Lymphoreplete Mice through Induction of Host Immunity

Gray Kueberuwa, Milena Kalaitsidou, Eleanor Cheadle, Robert Edward Hawkins, and David Edward Gilham

Supplementary Figure 1

A)

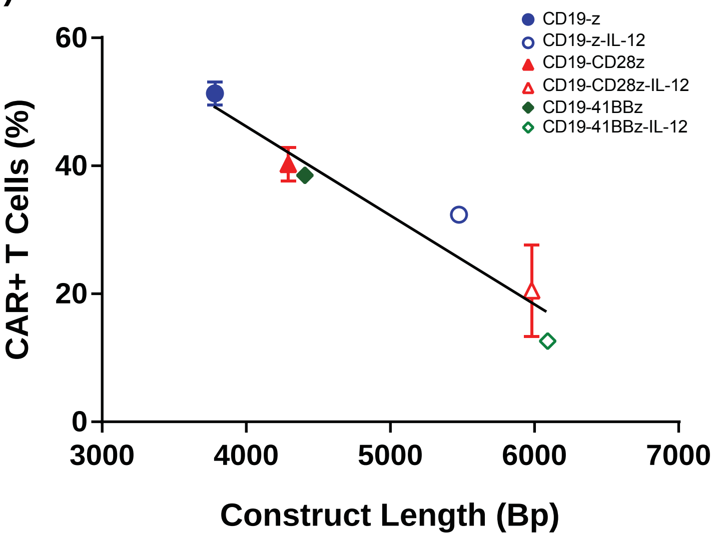

Supplementary Figure 2

A)

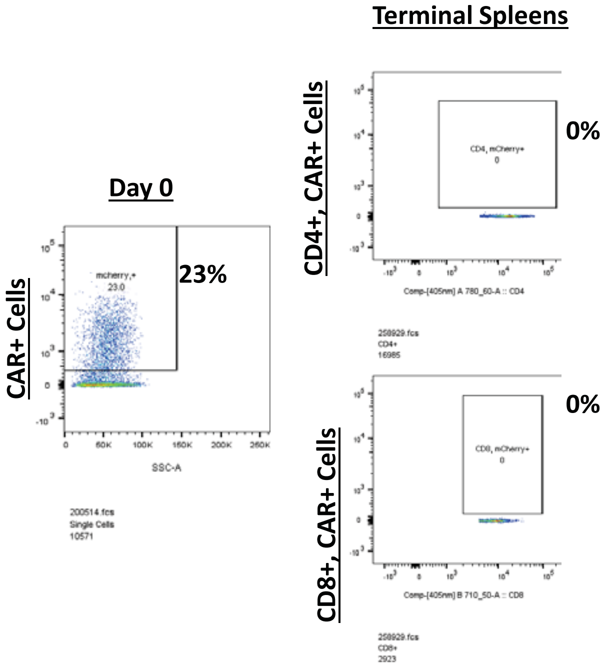

B)

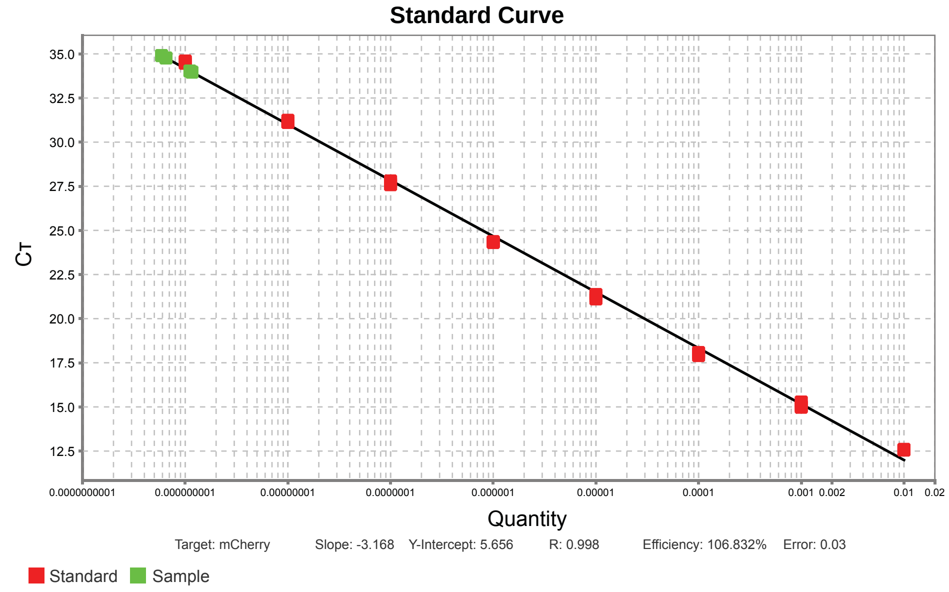

Supplement: Document S1. Figures S1 and S2 [file mmc1.pdf]
