# Supplementary material for: CD19 CAR T Cells Expressing IL-12 Eradicate Lymphoma in Fully Lymphoreplete Mice through Induction of Host Immunity
Source: Mol Ther Oncolytics. 2017 Dec 19;8:41–51. doi: 10.1016/j.omto.2017.12.003 (PMC5772011; doi:10.1016/j.omto.2017.12.003)

Molecular Therapy
Oncolytics
Original Article

CD19 CAR T Cells Expressing IL-12 Eradicate Lymphoma in Fully Lymphoreplete Mice through Induction of Host Immunity

Gray Kueberuwa,[1] Milena Kalaitsidou,[1] Eleanor Cheadle,[1] Robert Edward Hawkins,[1] and David Edward Gilham[1]

[1]Institute of Cancer Sciences, Manchester Cancer Research Centre Building, Wilmslow Road, Withington, Manchester M20 4QL, UK

Chimeric antigen receptor (CAR) T cell therapy represents a significant advancement in cancer therapy. Larger studies have shown ∼90% complete remission rates against chemoresistant and/or refractory CD19+ leukemia or lymphoma. Effective CAR T cell therapy is highly dependent on lymphodepleting preconditioning, which is achieved through chemotherapy or radiotherapy that carries with it significant toxicities. These can exclude patients of low performance status. In order to overcome the need for preconditioning, we constructed fully mouse first and second generation anti-murine CD19 CARs with or without interleukin-12 (IL-12) secretion. To test these CARs, we established a mouse model to reflect the human situation without preconditioning. Murine second generation CAR T cells expressing IL-12 were capable of eradicating established B cell lymphoma with a long-term survival rate of ∼25%. We believe this to be the first study in a truly lymphoreplete model. We provide evidence that IL-12-expressing CAR T cells not only directly kill target CD19+ cells, but also recruit host immune cells to an anti-cancer immune response. This finding is critical because lymphodepletion regimens required for the success of current CAR T cell technology eliminate host immune cells whose anti-cancer activity could otherwise be harnessed by strategies such as IL-12-secreting CAR T cells.

INTRODUCTION

Chimeric antigen receptor (CAR) T cell therapy represents a significant advancement in cancer therapy. Primarily, clinical success has been observed in the treatment of CD19+ hematological malignancies.[1] Clinical data of CD19 CAR T cell therapy to date mainly consists of a wide range of small studies showing safety and efficacy. These studies vary widely in recruitment criteria (including CD19+ cancer types, patient age, and treatment history), CAR structures, CAR T cell dose, methodology of cell production, and the use of preconditioning chemotherapy. Despite these extensive variations, results have largely been promising throughout.[2,3]

Larger studies assessing response rates have shown that second generation CD19 CARs with either CD28 or 41BB co-stimulatory domains are highly efficacious. CD19-41BBz CAR T cells led to 90% complete remissions (CRs) in children and adults with chemotherapy-resistant or refractory leukemia or lymphoma; 70% of these CRs were event free at 6 months.[4] CD19-CD28z CAR T cells also achieved an 88% complete response rate (CRR) in adult relapsed acute lymphoblastic leukemia (ALL).[5] These numbers are in comparison with 31%–44% CRs observed with standard of care salvage therapy after first relapse.[6] In the setting of chronic lymphocytic leukemia (CLL), data with a second generation CD19-41BBz CAR suggests lower overall response rates, reported at around 45%.[7] However, this is in comparison with 30% and 25% compete response rates in relapsed or refractory CLL observed with fludarabine, cyclophosphamide, and rituximab or pentostatin, cyclophosphamide, and rituximab combinations, respectively.[8,9]

Effective CAR T cell therapy is highly dependent on lymphodepleting preconditioning, which is achieved through chemotherapy agents and less frequently radiotherapy.[10–12] Moreover, increased intensity of pre-conditioning has been reported to correlate with the persistence and, therefore, therapeutic activity of adoptively transferred T cells.[13] A particular clinical study by Brentjens et al.[11] highlights the importance of preconditioning. The study included two cohorts of ALL and CLL patients, the first receiving no prior conditioning and the second receiving 1.5–3.0 g/m^2 cyclophosphamide preconditioning the day prior to receiving CD19CAR-CD28z T cells. Patients receiving cyclophosphamide showed greater CAR T cell persistence by flow cytometry, immunohistochemistry (IHC), and RT-PCR, as well as enhanced clinical activity. A second key study, carried out by Turtle et al.,[14] showed that preconditioning regimens including fludarabine were superior to those with cyclophosphamide alone, inducing 50% and 8% CRRs, respectively.

Studies so far suggest that preconditioning, which depletes endogenous lymphoid cells, improves CAR T cell therapy by removing competition for lymphoproliferative cytokines and space in lymphoid tissues.[15] In addition, there is evidence that activation of antigen-presenting cells (APCs),[16] depletion of Tregs,[17–19] and

Received 13 December 2017; accepted 15 December 2017;
https://doi.org/10.1016/j.omto.2017.12.003.

Correspondence: Gray Kueberuwa, Clinical and Experimental Immunotherapy Group, Institute of Cancer Sciences, MCRC Building, Wilmslow Road, Withington, Manchester M20 4QL, UK.
E-mail: gray.kueberuwa@manchester.ac.uk

This is an open access article under the CC BY license (http://creativecommons.org/licenses/by/4.0/).

reduction of tumor burden[20] all contribute to enhancement of adoptive T cell therapies by preconditioning regimens.

While preconditioning enhances CAR T cell therapy, agents used to achieve this carry with them significant toxicities.[21–24] Potential CAR T cell therapy patients must, therefore, be of an appropriate performance status to endure these toxicities. What is more, those that are eligible are susceptible to the complications associated with high doses of these drugs in addition to any side effects from the CAR T cell therapy itself or supportive interleukin-2 (IL-2), which is used in a minority of cases to enhance T cell proliferation and persistence *in vivo*.[25]

Overcoming the need for lymphodepletion as a requisite for effective CAR T cell therapy, as well as IL-2 cytokine support, would remove these toxicities from CAR T cell treatment regimens and allow the inclusion of patients in a less robust state. Patients able to tolerate associated toxicities would clearly also benefit from reduced discomfort. Furthermore, management of such toxicities is often costly due to the need for inpatient hospitalization and extensive monitoring and supportive care.

Pre-clinical models of CAR T cell therapy suffer from several problems. Systems assessing human tumor cells require the use of immune-deficient mice to allow engraftment of human tumors.[26] What is more, the administration of human T cells into mice requires even more severely immune-deficient mice and is complicated by the development of xenogeneic graft versus host disease, where human immune cells attack mouse cells.[27,28] In these systems, due to the lack of other immune cells, only direct effects of administered T cells can be observed.[29]

Syngeneic mouse models avoid these problems by using mouse tumors in mice of the same strain and also T cells from mice of the same strain. Although there may be variations between human and mouse tumors and immune systems, these systems allow cross-talk between immune cells to play a part in disease development and therapeutic mechanisms, which overall is more reflective of the human clinical situation than xenograft models.[30,31] We, therefore, utilized the syngeneic A20 B cell lymphoma model and CD19 targeting CARs of mouse genetic background (mCD19-CARs).

IL-12 is a cytokine that is produced by macrophages, dendritic cells (DCs), natural killer (NK) cells, and T cells that has a range of biological functions.[32,33] Primarily, it enhances interferon γ (IFNγ) production and cytotoxic effector functions of NK and T cells.[34] Additionally, it promotes a Th1 phenotype in CD4 cells, enhances antibody-dependent cell-mediated cytotoxicity (ADCC), and induces production of IP10, which is a chemoattractant to DCs, macrophages, NK cells, and T cells.[35] Together these actions have the potential to exert significant anti-tumor potency, which has been the subject of much investigation.[36,37]

Building on a previous report of IL-12 secretion from within CAR T cells allowing autocrine stimulation and significant engraftment with minimum lymphodepletion,[12] we present a fully lymphoreplete mouse model and assess the activity of IL-12-expressing CAR T cells and the importance of co-stimulatory molecules therein.

RESULTS

CD19 CAR T Cell Therapy Requires Lymphodepletion for Anti-cancer Efficacy

In lymphoreplete patients, endogenous T cells are much more abundant in number than adoptively transferred T cells. Adoptively transferred T cells are therefore outcompeted for binding of cytokines that induce proliferation, as well as physical space in lymphoid tissues; this leads to poor persistence and limited anti-cancer efficacy. Lymphodepletion, achieved through chemotherapy or radiotherapy, reduces endogenous lymphocyte numbers. This tips the balance of competition in favor of adoptively transferred T cells and allows efficient engraftment.

In BALB/c mice, with lymphodepletion via 5 Gy total body irradiation (TBI) prior to administration of CD19-z CAR T cells, dose dilution of CAR T cells shows that high numbers of both CD4 and CD8 CAR T cells persist in the circulation for 1 week when the administered dose is above 10^6 CAR T cells per mouse. Without prior lymphodepletion, circulating CAR T cells could not be detected (Figures 1A and 1B).

Having established that a dose of 10^6 CAR T cells with prior lymphodepletion leads to CAR T cells making up ~10%–30% of total circulating T cells after 1 week, we found that this persistence in the circulation was concomitant with eradication of systemic A20 B cell lymphoma expressing luciferase and GFP (A20.Luc.GFP). This was demonstrated by a reduction in tumor burden to background levels as measured by luminometry (Figure 1C) and a 100% survival rate at 100 days post T cell injection in mice receiving prior lymphodepletion compared with 16.7% in those that did not and 0% for saline-treated mice with or without lymphodepletion (Figure 1D).

Lymphoreplete B Cell Lymphoma Model

The A20.Luc.GFP systemic lymphoma model has very poor tumor take rates and extended periods of time for significant tumor burden and pathology to occur. A single dose of cyclophosphamide 1 day prior to delivery of A20.Luc.GFP cells drastically increases tumor take rate and decreases the time taken for systemic lymphoma to arise. In order to study the treatment of lymphoma in lymphoreplete mice, we sought to determine appropriate lymphodepletion prior to tumor cell administration that is high enough to induce an efficient tumor take rate, but is low enough to allow blood counts to fully recover before lethal tumor-related toxicities occur in mice. First, we compared two standard lymphodepletion methods, 5 Gy TBI and 200 mg/kg intravenous (i.v.) delivery of cyclophosphamide (Figures 2A and 2B). Results show a much more pronounced and prolonged depletion of lymphocytes and monocytes with 5 Gy TBI compared with 200 mg/kg cyclophosphamide. Importantly, with both 5 Gy TBI and 200 mg/kg cyclophosphamide, blood counts had not recovered to normal levels by 14 days (Figures 2A and 2B), by which time it begins to become necessary to sacrifice mice due to tumor-related

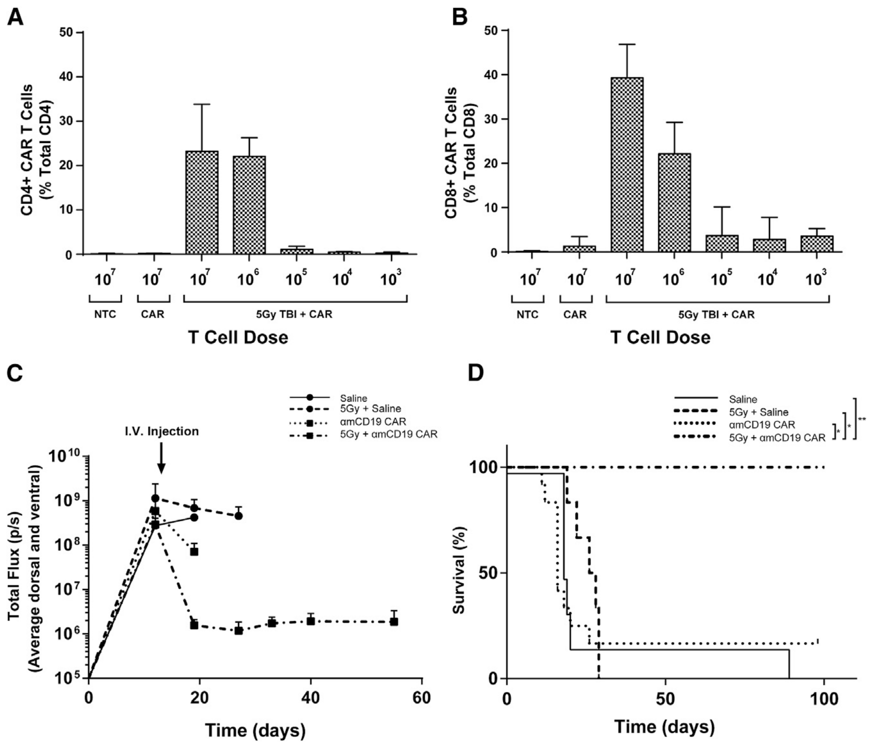

Figure 1. Successful CD19 CAR T Cell Therapy Requires Prior Lymphodepletion

Mice bearing established systemic A20.Luc.GFP lymphoma were given indicated T cell doses via i.v. injection. Preconditioned mice received 5 Gy TBI the day prior to T cells. Blood samples were collected via tail vein bleeds 7 days after T cell injection, and CAR T cell numbers were analyzed by flow cytometry. (A and B) The percentages of CAR T cells in total (A) $CD4^+$ and (B) $CD8^+$ circulating T cells are shown. Error bars show +SD. (C and D) Bioluminescence (C) and survival (D) of mice with established A20.Luc.GFP lymphoma treated with saline or 10^6 CAR T cells with or without 5 Gy TBI 1 day prior to i.v. injections. Statistical analysis was performed using log rank (Mantel-Cox) test. $^*p < 0.05$; $^{**}p < 0.01$. n = 6.

toxicities (data not shown). In contrast, reduction of the standard 200 mg/kg cyclophosphamide dose to 100 mg/kg did not lead to statistically significant reduction in blood counts over a period of 21 days (Figure 2C).

To investigate whether a reduced dose of cyclophosphamide could give efficient engraftment of A20.Luc.GFP lymphoma without significantly reducing blood counts, we gave mice 200, 100, or 50 mg/kg cyclophosphamide i.v. 1 day prior to 5×10^5 A20.Luc.GFP cells i.v. The dose level of cyclophosphamide correlated with tumor growth as measured by luminometry with all levels of cyclophosphamide leading to an increase in tumor growth over 42 days (Figures 3A and S1). Induction of lymphoma and survival of mice with 50 mg/kg cyclophosphamide was slow with just two-thirds of mice succumbing to tumor-related toxicity over 100 days. 200 and 100 mg/kg both led to 100% tumor take; however, the 100 mg/kg dose had a delayed onset of lymphoma and increased median survival (Figures 3A and 3B). Interestingly, tumor pathology was solely hind-limb paralysis (HLP; which occurs due to tumor invasion of the meninges) at the 200 mg/kg dose. At 100 mg/kg, HLP was observed at early time points, and at later time points solid tumor masses, usually in the peritoneum but arising anywhere throughout the body, were observed.

Due to 100% tumor take rate and lack of reduction in mouse blood counts at the 100 mg/kg dose level of cyclophosphamide, this was adopted as the standard dose for induction of A20.Luc.GFP lymphoma. In this disease model we could test CAR T cell therapy in a lymphoreplete setting.

CAR T Cells Expressing Murine IL-12

First and second generation anti-mouse CD19 CARs were engineered to express a single-chain murine IL-12. Anti-mouse CD19 CAR constructs were generated in the MP71 retroviral vector backbone and consisted of an mCherry marker gene under transcriptional control of the 5′ long terminal repeat (LTR) followed by the FMDV2A linker sequence for equimolar expression of the anti-mouse CD19 single-chain variable fragment (scFv) with the oncostatin M leader sequence for transport to the surface membrane. First generation vectors contained full-length murine CD3ζ immediately following the scFv, while second generation vectors contained full-length CD28 and 41BB co-stimulatory domains followed by murine CD3ζ starting from amino acids LRAK. For expression of single-chain murine IL-12 as described by Jiang et al.,[38] a porcine teschovirus 2A linker sequence was inserted directly downstream of CARs followed by IL-12 for equimolar expression (Figure 4A).

Retroviral transduction of mouse T cells displayed variable efficiency with CAR constructs encoding single-chain murine IL-12 having poorer transduction rates than non-IL-12-expressing counterparts (Figure 4B). The construct encoding αmCD19.m41BB.mCD3ζ.IL-12 (CD19-41BBz-IL-12) displayed the poorest rate of gene transfer, as measured by the proportion of mCherry-positive cells 3 days after transduction by flow cytometry (Figure 4B). An increase in the length of retroviral vector constructs correlated with a decrease in T cell transduction rate ($p = 0.0025$; $R^2 = 0.9192$; Figure S1), most likely because of inefficient genomic encapsidation during virus particle formation in producer cells.[39]

To assess the murine CD19-specific reactivity of CAR constructs, we assessed the ability to kill A20.Luc.GFP cells by luciferase protein presence following co-culture with CAR T cells. Assay conditions were optimized to display differential activity between CAR constructs. Results showed that upon 20-hr co-culture at a CAR T cell/target cell ratio of 1, CD19-CD28z-IL-12 and CD19-41BBz-IL-12

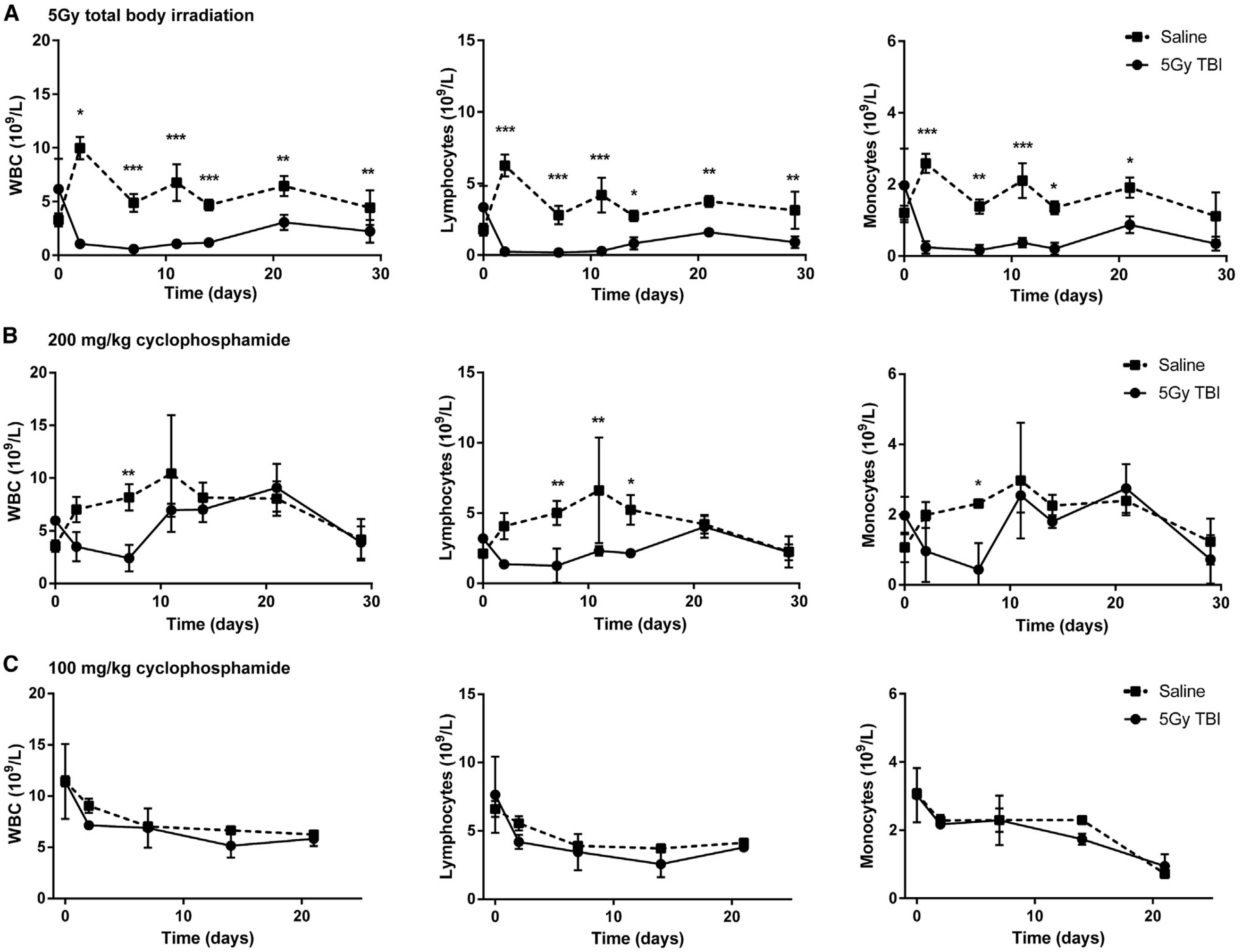

Figure 2. Longevity of Lymphodepletion

(A–C) Mice were given (A) 5 Gy TBI at 0.02 Gy/min, (B) 200 mg/kg cyclophosphamide i.p., or (C) 100 mg/kg cyclophosphamide i.p. 1 day prior to 5×10^5 A20.Luc.GFP cells i.v. Blood samples were collected at indicated times and blood counts determined using an automated counter. Error bars show ±SD. Statistical analysis was performed using two-way ANOVA. $^*p < 0.05$; $^{**}p < 0.01$; $^{***}p < 0.001$. n = 3.

CAR T cells demonstrated a 2.1- and a 2.5-fold enhancement in cytotoxicity, respectively, compared with non-IL-12-expressing counterparts CD19-28z and CD19-41BBz. In contrast, the cytotoxicity of first generation CAR T cells with no co-stimulation was not enhanced by IL-12 under these conditions (Figure 4C). Interestingly, CD19-41BBz CARs consistently showed poor cytotoxic activity *ex vivo*, although IL-12 co-expression enhanced cytotoxicity of CD19-41BBz-IL-12 CARs to levels similar to first generation or CD28 second generation CARs (with or without IL-12 co-expression). Longer co-culture periods generally led to a plateau in target cell numbers and, therefore, similar levels of target cell killing between CD19-z, CD19-z-IL-12, CD19-CD28z, and CD19-CD28z-IL-12 (data not shown).

As well as the ability of αmCD19-CAR T cell variants to kill target cells, IFNγ and IL-12 cytokine release was analyzed by ELISA following co-culture of CAR T cells with CD19+ A20.Luc.GFP cells. For vectors encoding IL-12, the presence of CD28 or 41BB co-stimulatory domains had an effect on the amount of IL-12 produced by CAR T cells. CD19-z-IL-12 CAR T cells produced 3.3×10^4 pg/mL, whereas CD19-CD28z-IL-12 CAR T cells produced 3.0-fold and CD19-41BBz-IL-12 CAR T cells produced 6.6-fold more than this, correlating co-stimulation with increased IL-12 production (Figure 4D). Notably, CD19-41BBz-IL-12 CAR T cells produced 2.2-fold more IL-12 than corresponding CD19-CD28z-IL-12 CAR T cells, perhaps suggesting synergy between 41BB and IL-12 signaling above that observed with CD28.

All CAR T cells expressing IL-12 produced increased levels of IFNγ compared with non-IL-12-expressing counterparts upon 20-hr incubation with target A20 cells (Figure 4E). CD19-z-IL-12 and

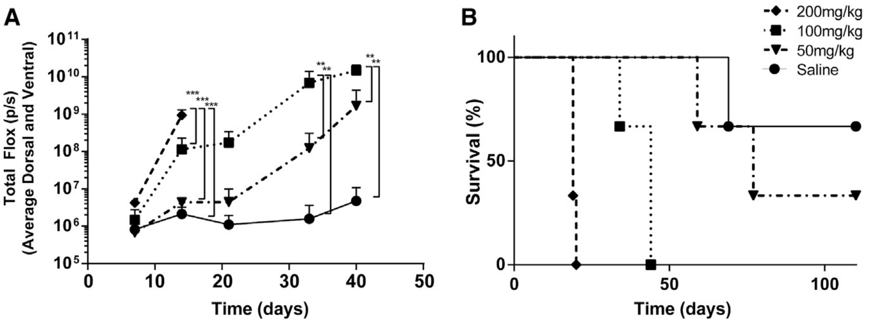

Figure 3. Cyclophosphamide Dose and Initiation of A20.Luc.GFP B Cell Lymphoma

Mice were given varying i.p. doses of cyclophosphamide 1 day prior to i.v. delivery of 5×10^5 A20.Luc.GFP cells the following day. (A and B) Increasing cyclophosphamide enhanced establishment of B cell lymphoma and shortened the time length of disease progression shown by (A) measurement of tumor burden by luminometry (error bars show +SD; statistical analysis was performed using two-way ANOVA; $^{**}p < 0.01$; $^{***}p < 0.001$) and (B) survival (n = 3).

CD19-41BBz-IL-12 CAR T cells produced similar levels, whereas CD19-CD28z-IL-12 CAR T cells produced ~2.8-fold more IFNγ, suggesting crossover between IFNγ and CD28 signaling.

CAR and CAR-IL-12 T Cells in Lymphoreplete Hosts

There are much data regarding increased effector cell functions and persistence of second generation CAR T cells compared with first generation CAR T cells in combination with prior lymphodepleting preconditioning.[2]

Previous work by Brentjens and colleagues[12] showed eradication of 10^6 EL4 tumor cells expressing human CD19 in transgenic C57BL6(mCD19$^{-/-}$hCD19$^{+/-}$) with a first generation anti-human CD19 CAR expressing murine IL-12. This important work shows that production of IL-12 from within first generation CAR T cells can overcome the need for prior lymphodepletion for effective CAR T cell therapy. Our data, however, suggest that the use of 250 mg/kg cyclophosphamide 72 hr prior to CAR T cell transfer means that these mice would not have been fully lymphoreplete at the time of treatment (Figure 2B); therefore, we looked to test our fully murine CAR T cells in fully lymphoreplete BALB/c mice. In addition, we looked to analyze eradication of B cell lymphoma in a more challenging situation of established disease, rather than 24 hr after tumor inoculation. In our test model we looked to assess whether murine CD28 or murine 41BB co-stimulatory domains could endow CAR T cells with enhanced persistence or effector cell functions in fully lymphoreplete hosts. In light of functional differences *ex vivo*, we also evaluated the effect of CD28 or 41BB co-stimulatory domains with constitutive murine IL-12 expression.

Lymphoreplete mice with A20 B cell lymphoma (as described above) were treated with αmCD19 CAR T cells, and circulating CAR T cell numbers were analyzed in blood samples collected 7 days post T cell administration by flow cytometry for the mCherry marker gene (Figures 5A and 5B). Circulating CAR T cells could only be detected in mice treated with CD19-41BBz or CD19-41BBz-IL-12 CAR T cells, and the levels detected were less than 0.2% of CD4$^+$ or CD8$^+$ T cells, much lower than levels seen with lymphodepletion before CAR T cell administration (Figures 1A and 1B). Significant numbers of CD4$^+$ CAR T cells were only detected in mice treated CD19-41BBz or CD19-41BBz-IL-12 CAR T cells, while circulating CD8$^+$ CAR T cells were only detected in mice treated with CD19-41BBz CAR T cells.

Tumor growth in mice was assessed by *in vivo* bioluminescence. Despite a very low level of circulating CAR T cells after 1 week, mice treated with CD19-CD28z-IL-12 or CD19-41BBz-IL-12 CAR T cells displayed a reduction in tumor growth at 3 weeks, followed by eradication of systemic B cell lymphoma in long-term survival of 26% and 22% of mice, respectively (Figures 5C and 5D). All other CAR constructs failed to induce long-term survival in any mice, although CD19-z-IL-12 extended survival beyond 100 days in ~11% of mice.

Significantly, in this and all subsequent experiments we did not observe any toxicity from CAR T cells expressing IL-12.

IL-12-Expressing CARs in Lymphoreplete Hosts Induce Robust Memory Immune Responses

To test for long-term persistence of CD19-CD28z-IL-12 and CD19-41BBzIL-12 CAR T cells in mice that successfully eradicated tumors, spleens were extracted and analyzed for the presence of CAR T cells by flow cytometry; no CAR T cells could be detected through this method (Figure S2A). In addition, qPCR for the detection of the mCherry marker gene, with a sensitivity of 15 genomes/well, was used to test for the persistence of CAR T cells. This method also failed to detect any residual input CAR T cells in surviving CAR-IL-12-treated mice with DNA from ~8 mg of spleen tissue, which equates to 1.6×10^6 genomes/well (Figure S2B).

Despite the absence of CAR T cells, incubation of splenocytes from long-term survivor CAR-IL-12-treated (C12T) mice with A20 tumor cells showed the presence of reactive T cells by IFNγ enzyme-linked immunospot (ELISpot) (Figure 6A). In addition, co-culture of splenocytes with A20 cells revealed modest, but significant cytotoxicity against tumor cells compared with splenocytes from tumor-naive mice (Figure 6B). Together, these data suggest subsidence of adoptively transferred CAR T cells *in vivo* and induction of anti-tumor immunity exerted by the host immune system causing clearance of systemic lymphoma.

To assess the anti-cancer potency of immune cells in the spleens of C12T mice, we adoptively transferred splenocytes to syngeneic

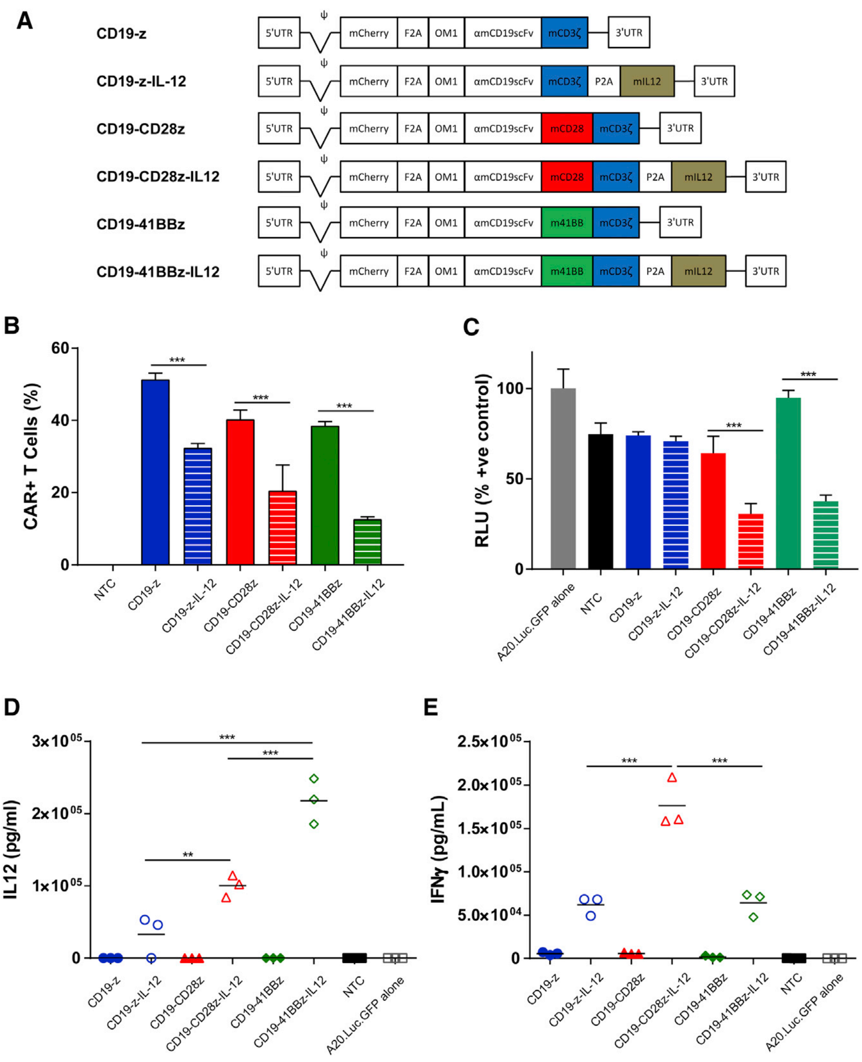

Figure 4. CD19 CAR Constructs

(A) Genetic maps of retroviral vectors encoding CD19 targeting CARs with variations in co-stimulatory domains and constituent expression of murine IL-12. (B) Mouse T cells were transduced with retroviral constructs, and transduction efficiency was determined by assessing the numbers of CAR T cells 3 days post-transduction by flow cytometry (n = 3). (C–E) CAR T cells were incubated at a ratio of 1:1 with A20.Luc.GFP cells, and (C) luminometry was used to assess the cytotoxicity after 20-hr co-culture (n = 4), while ELISAs after 40-hr co-culture were used to measure (D) IL-12 and (E) IFNγ release (n = 3). Error bars show ±SD. Statistical analysis was performed using one-way ANOVAs. $^{**}p < 0.01$; $^{***}p < 0.001$.

BALB/c-severe combined immunodeficiency (SCID) mice that lack lymphocytes of their own, bearing established A20.Luc.GFP systemic lymphoma. Upon confirmation of systemic tumor burden by *in vivo* bioluminescence, splenocytes from C12T mice that had eradicated the same tumor type or splenocytes from non-treated control mice were adoptively transferred. Analysis of tumor burden through luminometry showed an uncontrolled increase in tumor growth in mice that were treated with control splenocytes. Mice receiving splenocytes from C12T mice displayed a similar initial rate of tumor growth, followed by eradication of lymphoma in 80% of mice (Figure 6C). Tumor clearance as indicated by bioluminescence was concomitant with 80% survival at 100 days (Figure 6D). CD8 and CD4 T cells seem to co-operate to clear tumors upon adoptive transfer as depletion of CD8 cells from C12T populations led to a reduced survival advantage; however, this was still protective in comparison with control splenocytes (Figure 6E).

CAR-IL-12 T Cells Induce Epitope Spreading

CD19-CD28z-IL-12 or CD19-41BBz-IL-12 CAR T cells could eradicate systemic lymphoma and induced robust memory responses capable of clearing established lymphoma in BALB/c SCID mice upon adoptive transfer. However, it is noted that the tumor cell line used in the above studies expressed luciferase and GFP reporter genes; foreign proteins that are not representative of the leukemic situation that may be the subject of immune attack.

To assess the reactivity of immune cells from C12T mice against the original CD19 target antigen, luciferase or GFP, we analyzed the IFNγ release upon culture of splenocytes with A20, A20.GFP, or A20.Luc.GFP cell lines. No significant IFNγ release was observed, suggesting that clearance of tumors from these mice is not highly dependent on luciferase or GFP (Figure 7A). The amount of interferon released during these co-culture reactions, however, was very low and not consistent with the strong anti-tumor activity observed *in vivo*.

To assess whether transgenes were required for tumor clearance, we established systemic leukemia with A20 cells lacking any transgene, and mice were treated with CD19-CD28z-IL-12 CAR T cells. Results showed that treatment achieved a long-term survival rate of ∼36% (Figure 7B), comparable with systemic lymphoma expressing luciferase and GFP (Figure 5D). These data strongly suggest that CAR T cells expressing IL-12 can induce epitope spreading to tumor-associated antigens, and this can eradicate established lymphoma without the need for lymphodepleting preconditioning.

BALB/c SCID mice that had cleared systemic A20.Luc.GFP tumors upon receipt of adoptively transferred C12T splenocytes were re-challenged with A20 cells lacking luciferase or GFP, to further assess the contribution of reporter genes to tumor protection. Results showed

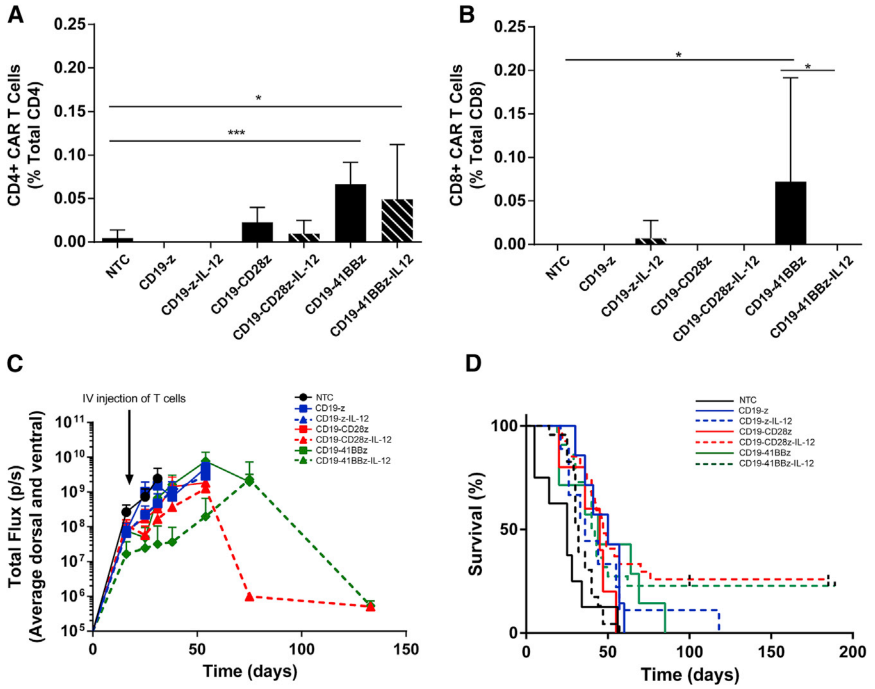

Figure 5. CD19 CARs Expressing IL-12 Are Capable of Eradication of Established Lymphoma in Lymphoreplete Mice

Lymphoreplete mice bearing established A20.Luc.GFP tumors were administered T cells via i.v. injection. (A and B) Peripheral blood was collected via tail vein puncture, and the numbers of (A) $CD4^+$ and (B) $CD8^+$ CAR T cells in the circulation were quantified by flow cytometry. Error bars show +SD (n = 9). Statistical analysis was performed using one-way ANOVA. Significant difference from non-treated control (NTC) or matched vector with IL-12 expression is shown. $^*p < 0.05$; $^{***}p < 0.001$. (C) Tumor growth was monitored by i.p. injection of luciferin and luminometry analysis. Error bars show +SEM (n = 9). (D) Survival analysis up to 189 days (n = 41–50).

that median survival of mice was extended about 4-fold (from 29 to 88 days) in comparison with naive mice (Figure 7C). This is indicative of immune response to proteins other than luciferase or GFP; however, the response was insufficient to prevent these mice from succumbing to lymphoma.

DISCUSSION

CAR T cell therapy to treat $CD19^+$ hematological malignancies has displayed great success in the clinic. One aspect of this therapy, however, is the requirement of toxic pre-conditioning regimens for therapeutic success. This toxicity, while acceptable for most patients, excludes those of poor performance status. In addition, inpatient care and close monitoring of patients inflates the cost of CAR T cell therapy.

Here, we developed a syngeneic mouse model of established B cell lymphoma validated to be fully lymphoreplete before treatment with CD19 CAR T cells. In this model, we showed that either first generation or second generation CD28 or 41BB CD19-CAR T cells were an ineffectual treatment. Second generation CD28 or 41BB CAR T cells expressing IL-12, however, led to eradication of established lymphoma and long-term survival in about a quarter of mice.

Long-term surviving mice did not show any evidence of long-term persisting CAR-T cells by either flow cytometry or qPCR. Despite this, they exhibited protection from tumor re-challenge. In addition, adoptive transfer of splenocytes from long-term surviving mice into BALB/c SCID mice bearing established B cell lymphoma led to tumor eradication. Together, these data suggest robust and long-lasting anti-tumor immune responses carried out by the host immune system, but induced by CAR T cells expressing IL-12 before their subsidence by 1 week after administration.

The mechanism of CAR T cell therapy is through direct killing of cells expressing target antigen by T cells expressing the CAR. It is possible that the inflammatory environment created by such tumor cell killing can lead to priming of immune responses against additional target antigens present on tumor cells.[40,41] IL-12 induces the production of multiple inflammatory cytokines including granulocyte-macrophage colony-stimulating factor (GM-CSF), tumor necrosis factor alpha (TNF-α), IL-6, IL-8, IL-15, and IL-18; activates NK cells and DCs; and enhances the cytotoxicity of T cells.[33,42–44] We hypothesize that IL-12 secreted from T cells overcomes immune suppression mediated by tumor cells and provides a conducive environment for epitope spreading, where immune cells diversify to attack multiple targets in addition to the original antigen, in this case, tumor-associated antigens.

These results are highly relevant because there have been several cases of immune escape of tumors from CD19 CAR T cell therapy where $CD19^+$ malignancies recur as $CD19^-$ populations. A CAR T cell therapy that induces epitope spreading, and therefore immune responses against several tumor-associated antigens, would reduce this phenomenon. What is more, depletion of existing immune cells with pre-conditioning regimens prior to administration of CAR T cells with an immune-stimulating agent may be obstructive to this desired epitope spreading.

In a similar fashion to other immune-based therapies in the clinic, we saw long-term survival in a proportion of subjects, but no extended survival or tumor eradication in others. Elements that determine therapeutic success remain elusive, and further work is required to elucidate causal factors and investigate how they can

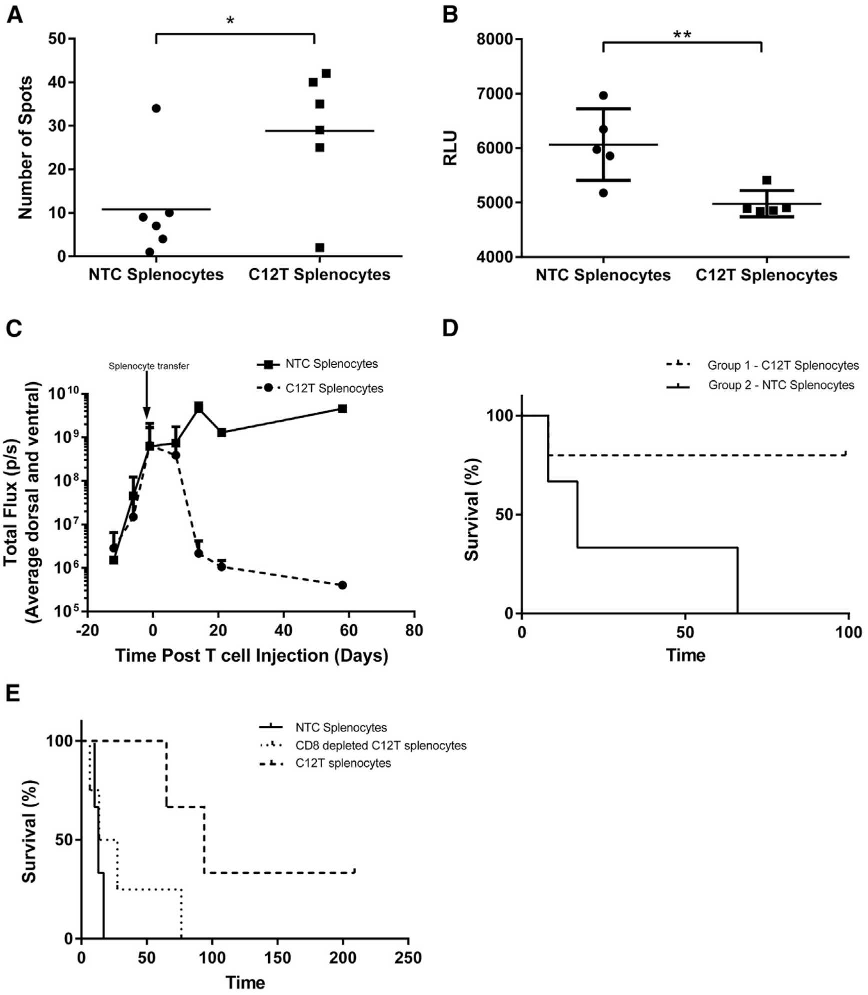

Figure 6. CD19 CARs Expressing IL-12 Induce Robust, Long-Lasting Anti-tumor Immune Responses

Mice that had A20.Luc.GFP lymphoma and were treated with CAR-IL-12 T cells that survived beyond 100 days had spleens harvested. Splenocytes were incubated with A20.Luc.GFP cells, and (A) ELISpot analysis was used to determine the frequency of reactive cells (splenocyte:A20 ratio = 1:1) (n = 6). (B) The cytotoxic activity of splenocytes toward A20.Luc.GFP cells was measured by 40-hr luciferase assay (splenocyte:A20 ratio = 50:1) (n = 6). (C and D) BALB/c SCID mice bearing established A20.Luc.GFP tumors received 1.8×10^7 splenocytes i.v., and tumor growth (C) and survival (D) were monitored (n = 5). (E) 1.2×10^7 total splenocytes were either directly administered or subjected to depletion of CD8 T cells before administration to BALB/c SCID mice bearing established systemic A20.Luc.GFP lymphoma, and survival was monitored (n = 4). $^*p < 0.05$; $^{**}p < 0.01$.

be overcome. The model developed here will provide a base for this moving forward.

A concern for the use of IL-12 as an anti-cancer therapy is toxicity, which was widely observed in early clinical investigations and also more recently with tumor-infiltrating lymphocytes against metastatic melanoma engineered to secrete IL-12 *in vivo*.[45] The use of pre-conditioning in previous studies, however, leads to much increased levels of engraftment compared with those seen in this study. Whether similar toxicities are observed without preconditioning will be of upmost importance.

In conclusion, we show here that production of IL-12 from second generation CD19 CAR T cells can eradicate established systemic lymphoma without the need for prior lymphodepletion. The mechanism of action of tumor eradication appears to be through a combination of direct cell killing and the induction of an anti-cancer immune response from existing host immune cells against tumor-associated antigens. This epitope spreading could be key in the advancement of CAR T cell therapy and reduction of immune escape by tumor cells.

MATERIALS AND METHODS

Cell Lines and Culture

PlatE cells (Cell Biolabs, USA) were maintained in DMEM, 10% fetal calf serum (FCS), and 5 mM glutamine. A20 cells were modified to express luciferase and GFP or GFP alone using the rkat retroviral vector prkat.luciferase.IRES.GFP or prkat.GFP derivative.[46]

A20 cells were cultured in RPMI 1640, 10% FCS, penicillin, streptomycin, glutamine, 4-(2-hydroxyethyl)-1-piperazineethanesulfonic acid (HEPES), and β-mercaptoethanol.

Retroviral Constructs

An MP71 retroviral vector encoding 1D3, an scFv against mouse CD19, and a truncated human CD34 extracellular domain were used as a template.[47] tCD34 was replaced with mCherry to reduce immunogenicity. Full-length murine CD28 and 41BB were synthesized and inserted in-frame between 1D3 and mCD3ζ to create murine second generation CARs. Single-chain, secreted murine IL-12 was synthesized downstream of a porcine teschovirus 2A site and inserted in-frame, directly downstream of the murine CD3ζ signaling domain in first and both second generation CAR vectors.

T Cell Transduction

PlatE cells were transfected with pclEco and MP71 retroviral constructs encoding CARs, and viral supernatant was collected and passed through a 0.45-μm filter (Appleton Woods, UK) 48 and 72 hr post-transfection before immediate use. Mouse splenocytes were transduced with retrovirus after activation overnight as previously described.[48] In

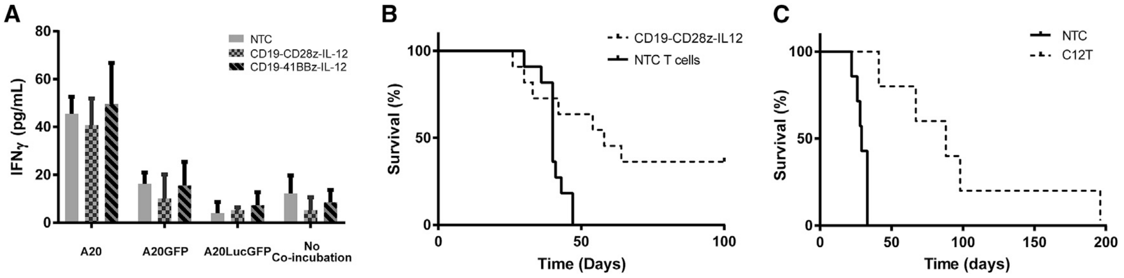

Figure 7. Tumor Clearance Is Not through Immune Attack of Reporter Proteins

Mice that had A20.Luc.GFP lymphoma and were treated with CAR-IL-12 T cells that survived beyond 100 days had spleens harvested. (A) Total splenocytes were incubated with 1×10^5 A20, A20.GFP, or A20.Luc.GFP cells for 48 hr, and IFNγ release was measured by ELISA (n = 4). (B) Lymphoreplete mice bearing established A20 tumors were administered CD19-CD28z-IL-12 CAR T cells or NTC T cells via i.v. injection, and survival was monitored. (n = 11). (C) Mice that had A20.Luc.GFP lymphoma and were treated with CAR-IL12 T cells that survived beyond 100 days were re-challenged with A20 lymphoma cells, and survival was monitored compared with naive mice (n = 7).

brief, splenocytes were incubated with anti-CD3 and anti-CD28 antibody (30 ng/mL) overnight. Splenocytes and transduced T cells were cultured in 100 U of human IL-2 and 2 ng/mL murine IL-7.

Ex Vivo Functionality Assays

CD19 CAR T cells were cultured with syngeneic target CD19$^+$ tumor cells modified to express luciferase in U-bottom 96-well plates at indicated ratios overnight. Following co-culture, plates were centrifuged at 500 × *g* for 5 min and supernatant collected for analysis of murine IFNγ (2B Scientific, UK) and murine IL-12p70 (eBioscience, USA) by ELISA according to manufacturers' protocols. Target cell killing was analyzed by resuspension of cell pellets in PBS containing luciferin, incubation for 10 min at 37°C, and use of a Lumistart Omega luminometer (BMG Labtech, Germany).

Flow Cytometry

T cells were stained with CD8-BV711, CD4-BV786, and ZombieUV (BioLegend) according to the manufacturer's protocol and analyzed on the same day with an LSR Fortessa X20 using mCherry fluorescence as a marker of CAR expression.

Mice and *In Vivo* Model

BALB/C mice were treated with cyclophosphamide a day before i.v. delivery of 5×10^5 syngeneic A20 B cell lymphoma cells expressing luciferase and GFP. For lymphoreplete tumor studies, mice were treated with 100 mg/kg cyclophosphamide, then A20 cells 24 hr later. 17 days later, when blood counts had reached normal levels and systemic lymphoma had been established, mice received a single dose of CAR T cells. Tumor burden was monitored by intraperitoneal (i.p.) injection of luciferin and imaging using an *in vivo* imaging system (Perkin Elmer, USA). For A20 lymphoma with no transgene, mice received 5×10^4 A20 cells i.v. and therapeutic CAR T cells 7 days postinjection. Circulating numbers of CAR T cells were determined by tail vein bleeds, antibody staining, and flow cytometry. All experiments were conducted under the auspices of the Animals (Scientific Procedures) Act 1986 and under UK Coordinating Committee for Cancer Research guidelines. All animal studies were conducted at the Manchester Cancer Institute, which was approved by the CRUK-Manchester institute local animal welfare & ethics review body (CRUK-MI AWERB).

Statistical Analysis

GraphPad Prism 5 software (GraphPad Software) was used for statistical analysis. Test used and error bar properties are described in the figure legends. Survival curves begin at the day of i.v. CAR T cell or splenocyte injection. Kaplan–Meier curves are either representative of at least three experimental repeats or the amalgamation of data from three experiments.

SUPPLEMENTAL INFORMATION

Supplemental Information includes two figures and can be found with this article online at https://doi.org/10.1016/j.omto.2017.12.003.

AUTHOR CONTRIBUTIONS

G.K. designed and performed research, analyzed data, and wrote the manuscript. M.K. and E.C. contributed to proofreading. D.E.G. and R.E.H. provided expert advice.

CONFLICTS OF INTEREST

The authors declare no conflict of interest.

ACKNOWLEDGMENTS

We would like to thank Bloodwise for funding this research (grant 13031) and the CRUK Manchester biological resource unit (particularly Diane Beeston, Adam Freestone, and Suzanne Kelly) imaging and cytometry and molecular biology core facilities for supporting this work.

REFERENCES

1. Wang, Z., Guo, Y., and Han, W. (2017). Current status and perspectives of chimeric antigen receptor modified T cells for cancer treatment. Protein Cell *8*, 896–925.
2. Johnson, L.A., and June, C.H. (2017). Driving gene-engineered T cell immunotherapy of cancer. Cell Res. *27*, 38–58.
3. Davila, M.L., Sauter, C., and Brentjens, R. (2015). CD19-targeted T cells for hematologic malignancies: clinical experience to date. Cancer J. *21*, 470–474.

4. Maude, S.L., Frey, N., Shaw, P.A., Aplenc, R., Barrett, D.M., Bunin, N.J., Chew, A., Gonzalez, V.E., Zheng, Z., Lacey, S.F., et al. (2014). Chimeric antigen receptor T cells for sustained remissions in leukemia. N. Engl. J. Med. *371*, 1507–1517.

5. Davila, M.L., Riviere, I., Wang, X., Bartido, S., Park, J., Curran, K., Chung, S.S., Stefanski, J., Borquez-Ojeda, O., Olszewska, M., et al. (2014). Efficacy and toxicity management of 19-28z CAR T cell therapy in B cell acute lymphoblastic leukemia. Sci. Transl. Med. *6*, 224ra25.

6. Gökbuget, N., Stanze, D., Beck, J., Diedrich, H., Horst, H.-A., Hüttmann, A., Kobbe, G., Kreuzer, K.A., Leimer, L., Reichle, A., et al.; German Multicenter Study Group for Adult Acute Lymphoblastic Leukemia (2012). Outcome of relapsed adult lymphoblastic leukemia depends on response to salvage chemotherapy, prognostic factors, and performance of stem cell transplantation. Blood *120*, 2032–2041.

7. Mato, A., and Porter, D.L. (2015). A drive through cellular therapy for CLL in 2015: allogeneic cell transplantation and CARs. Blood *126*, 478–485.

8. Badoux, X.C., Keating, M.J., Wang, X., O'Brien, S.M., Ferrajoli, A., Faderl, S., Burger, J., Koller, C., Lerner, S., Kantarjian, H., and Wierda, W.G. (2011). Fludarabine, cyclophosphamide, and rituximab chemoimmunotherapy is highly effective treatment for relapsed patients with CLL. Blood *117*, 3016–3024.

9. Lamanna, N., Kalaycio, M., Maslak, P., Jurcic, J.G., Heaney, M., Brentjens, R., Zelenetz, A.D., Horgan, D., Gencarelli, A., Panageas, K.S., et al. (2006). Pentostatin, cyclophosphamide, and rituximab is an active, well-tolerated regimen for patients with previously treated chronic lymphocytic leukemia. J. Clin. Oncol. *24*, 1575–1581.

10. Brentjens, R.J., Riviere, I., Park, J., Davilla, M., Wang, X., Yeh, R., Lamanna, N., Frattini, M.G., and Sadelain, M. (2011). Lymphodepletion and tumor burden govern clinical responses in patients with B-cell malignancies treated with autologous, CD19-targeted T cells. J. Clin. Oncol. *29* (*Suppl 15*), 2534.

11. Brentjens, R.J., Rivière, I., Park, J.H., Davila, M.L., Wang, X., Stefanski, J., Taylor, C., Yeh, R., Bartido, S., Borquez-Ojeda, O., et al. (2011). Safety and persistence of adoptively transferred autologous CD19-targeted T cells in patients with relapsed or chemotherapy refractory B-cell leukemias. Blood *118*, 4817–4828.

12. Pegram, H.J., Lee, J.C., Hayman, E.G., Imperato, G.H., Tedder, T.F., Sadelain, M., and Brentjens, R.J. (2012). Tumor-targeted T cells modified to secrete IL-12 eradicate systemic tumors without need for prior conditioning. Blood *119*, 4133–4141.

13. Hershkovitz, L., Schachter, J., Treves, A.J., and Besser, M.J. (2010). Focus on adoptive T cell transfer trials in melanoma. Clin. Dev. Immunol. *2010*, 260267.

14. Turtle, C.J., Hanafi, L.-A., Berger, C., Hudecek, M., Pender, B., Robinson, E., et al. (2016). Immunotherapy of non-Hodgkin's lymphoma with a defined ratio of CD8+ and CD4+ CD19-specific chimeric antigen receptor-modified T cells. Sci. Transl. Med. *8*, 355ra116.

15. Gattinoni, L., Finkelstein, S.E., Klebanoff, C.A., Antony, P.A., Palmer, D.C., Spiess, P.J., Hwang, L.N., Yu, Z., Wrzesinski, C., Heimann, D.M., et al. (2005). Removal of homeostatic cytokine sinks by lymphodepletion enhances the efficacy of adoptively transferred tumor-specific CD8+ T cells. J. Exp. Med. *202*, 907–912.

16. Kang, T.H., Mao, C.-P., Lee, S.Y., Chen, A., Lee, J.-H., Kim, T.W., Alvarez, R.D., Roden, R.B., Pardoll, D., Hung, C.F., and Wu, T.C. (2013). Chemotherapy acts as an adjuvant to convert the tumor microenvironment into a highly permissive state for vaccination-induced antitumor immunity. Cancer Res. *73*, 2493–2504.

17. Wallen, H., Thompson, J.A., Reilly, J.Z., Rodmyre, R.M., Cao, J., and Yee, C. (2009). Fludarabine modulates immune response and extends in vivo survival of adoptively transferred CD8 T cells in patients with metastatic melanoma. PLoS ONE *4*, e4749.

18. Beyer, M., Kochanek, M., Darabi, K., Popov, A., Jensen, M., Endl, E., Knolle, P.A., Thomas, R.K., von Bergwelt-Baildon, M., Debey, S., et al. (2005). Reduced frequencies and suppressive function of CD4+CD25hi regulatory T cells in patients with chronic lymphocytic leukemia after therapy with fludarabine. Blood *106*, 2018–2025.

19. Curiel, T.J., Coukos, G., Zou, L., Alvarez, X., Cheng, P., Mottram, P., Evdemon-Hogan, M., Conejo-Garcia, J.R., Zhang, L., Burow, M., et al. (2004). Specific recruitment of regulatory T cells in ovarian carcinoma fosters immune privilege and predicts reduced survival. Nat. Med. *10*, 942–949.

20. Park, J.H., Rivière, I., Wang, X., Stefanski, J., He, Q., Taylor, C., Olszewska, M., Wasielewska, T., Bartido, S., Davila, M.L., et al. (2015). Impact of the conditioning chemotherapy on outcomes in adoptive T cell therapy: results from a phase I clinical trial of autologous CD19-targeted T cells for patients with relapsed CLL. Blood *120*, 1797.

21. Dudley, M.E., Wunderlich, J.R., Yang, J.C., Sherry, R.M., Topalian, S.L., Restifo, N.P., Royal, R.E., Kammula, U., White, D.E., Mavroukakis, S.A., et al. (2005). Adoptive cell transfer therapy following non-myeloablative but lymphodepleting chemotherapy for the treatment of patients with refractory metastatic melanoma. J. Clin. Oncol. *23*, 2346–2357.

22. Gunturu, K.S., Meehan, K.R., Mackenzie, T.A., Crocenzi, T.S., McDermott, D., Usherwood, E.J., Margolin, K.A., Crosby, N.A., Atkins, M.B., Turk, M.J., et al. (2010). Cytokine working group study of lymphodepleting chemotherapy, interleukin-2, and granulocyte-macrophage colony-stimulating factor in patients with metastatic melanoma: clinical outcomes and peripheral-blood cell recovery. J. Clin. Oncol. *28*, 1196–1202.

23. Bouvet, E., Borel, C., Obéric, L., Compaci, G., Cazin, B., Michallet, A.-S., Laurent, G., and Ysebaert, L. (2013). Impact of dose intensity on outcome of fludarabine, cyclophosphamide, and rituximab regimen given in the first-line therapy for chronic lymphocytic leukemia. Haematologica *98*, 65–70.

24. Eve, H.E., Linch, D., Qian, W., Ross, M., Seymour, J.F., Smith, P., Stevens, L., and Rule, S.A. (2009). Toxicity of fludarabine and cyclophosphamide with or without rituximab as initial therapy for patients with previously untreated mantle cell lymphoma: results of a randomised phase II study. Leuk. Lymphoma *50*, 211–215.

25. Acquavella, N., Kluger, H., Rhee, J., Farber, L., Tara, H., Ariyan, S., Narayan, D., Kelly, W., and Sznol, M. (2008). Toxicity and activity of a twice daily high-dose bolus interleukin 2 regimen in patients with metastatic melanoma and metastatic renal cell cancer. J. Immunother. *31*, 569–576.

26. Richmond, A., and Su, Y. (2008). Mouse xenograft models vs GEM models for human cancer therapeutics. Dis. Model. Mech. *1*, 78–82.

27. Zitvogel, L., Pitt, J.M., Daillère, R., Smyth, M.J., and Kroemer, G. (2016). Mouse models in oncoimmunology. Nat. Rev. Cancer *16*, 759–773.

28. Alcantar-Orozco, E.M., Gornall, H., Baldan, V., Hawkins, R.E., and Gilham, D.E. (2013). Potential limitations of the NSG humanized mouse as a model system to optimize engineered human T cell therapy for cancer. Hum. Gene Ther. Methods *24*, 310–320.

29. Kalaitsidou, M., Kueberuwa, G., Schütt, A., and Gilham, D.E. (2015). CAR T-cell therapy: toxicity and the relevance of preclinical models. Immunotherapy *7*, 487–497.

30. Mosely, S.I., Prime, J.E., Sainson, R.C., Koopmann, J.O., Wang, D.Y., Greenawalt, D.M., Ahdesmaki, M.J., Leyland, R., Mullins, S., Pacelli, L., et al. (2016). Rational selection of syngeneic preclinical tumor models for immunotherapeutic drug discovery. Cancer Immunol. Res. *5*, 29–41.

31. Sanmamed, M.F., Chester, C., Melero, I., and Kohrt, H. (2016). Defining the optimal murine models to investigate immune checkpoint blockers and their combination with other immunotherapies. Ann. Oncol. *27*, 1190–1198.

32. Trinchieri, G. (1995). Interleukin-12: a proinflammatory cytokine with immunoregulatory functions that bridge innate resistance and antigen-specific adaptive immunity. Annu. Rev. Immunol. *13*, 251–276.

33. Liu, J., Cao, S., Kim, S., Chung, E.Y., Homma, Y., Guan, X., Jimenez, V., and Ma, X. (2005). Interleukin-12: an update on its immunological activities, signaling and regulation of gene expression. Curr. Immunol. Rev. *1*, 119–137.

34. Otani, T., Nakamura, S., Toki, M., Motoda, R., Kurimoto, M., and Orita, K. (1999). Identification of IFN-γ-producing cells in IL-12/IL-18-treated mice. Cell. Immunol. *198*, 111–119.

35. Dufour, J.H., Dziejman, M., Liu, M.T., Leung, J.H., Lane, T.E., and Luster, A.D. (2002). IFN-γ-inducible protein 10 (IP-10; CXCL10)-deficient mice reveal a role for IP-10 in effector T cell generation and trafficking. J. Immunol. *168*, 3195–3204.

36. Lasek, W., Zagożdżon, R., and Jakobisiak, M. (2014). Interleukin 12: still a promising candidate for tumor immunotherapy? Cancer Immunol. Immunother. *63*, 419–435.

37. Lu, X. (2017). Impact of IL-12 in cancer. Curr. Cancer Drug Targets *17*, 682–697.

38. Jiang, C., Magee, D.M., and Cox, R.A. (1999). Construction of a single-chain interleukin-12-expressing retroviral vector and its application in cytokine gene therapy against experimental coccidioidomycosis. Infect. Immun. *67*, 2996–3001.

39. Kumar, M., Keller, B., Makalou, N., and Sutton, R.E. (2001). Systematic determination of the packaging limit of lentiviral vectors. Hum. Gene Ther. *12*, 1893–1905.

40. Rabinovich, G.A., Gabrilovich, D., and Sotomayor, E.M. (2007). Immunosuppressive strategies that are mediated by tumor cells. Annu. Rev. Immunol. *25*, 267–296.

41. Daniels, G.A., Sanchez-Perez, L., Diaz, R.M., Kottke, T., Thompson, J., Lai, M., Gough, M., Karim, M., Bushell, A., Chong, H., et al. (2004). A simple method to cure established tumors by inflammatory killing of normal cells. Nat. Biotechnol. *22*, 1125–1132.

42. Robertson, M.J., Sr., Soiffer, R.J., Wolf, S.F., Manley, T.J., Donahue, C., Young, D., Herrmann, S.H., and Ritz, J. (1992). Response of human natural killer (NK) cells to NK cell stimulatory factor (NKSF): cytolytic activity and proliferation of NK cells are differentially regulated by NKSF. J. Exp. Med. *175*, 779–788.

43. Portielje, J.E.A., Lamers, C.H.J., Kruit, W.H.J., Sparreboom, A., Bolhuis, R.L.H., Stoter, G., Huber, C., and Gratama, J.W. (2003). Repeated administrations of interleukin (IL)-12 are associated with persistently elevated plasma levels of IL-10 and declining IFN-γ, tumor necrosis factor-α, IL-6, and IL-8 responses. Clin. Cancer Res. *9*, 76–83.

44. Tatsumi, T., Takehara, T., Kanto, T., Miyagi, T., Kuzushita, N., Sugimoto, Y., Jinushi, M., Kasahara, A., Sasaki, Y., Hori, M., and Hayashi, N. (2001). Administration of interleukin-12 enhances the therapeutic efficacy of dendritic cell-based tumor vaccines in mouse hepatocellular carcinoma. Cancer Res. *61*, 7563–7567.

45. Lasek, W., and Zagozdzon, R. (2016). Clinical trials with IL-12 in cancer immunotherapy. In Interleukin 12: Antitumor Activity and Immunotherapeutic Potential in Oncology, W. Lasek and R. Zagozdzon, eds. (Springer International Publishing), pp. 43–75.

46. Finer, M.H., Dull, T.J., Qin, L., Farson, D., and Roberts, M.R. (1994). kat: a high-efficiency retroviral transduction system for primary human T lymphocytes. Blood *83*, 43–50.

47. Engels, B., Cam, H., Schüler, T., Indraccolo, S., Gladow, M., Baum, C., Blankenstein, T., and Uckert, W. (2003). Retroviral vectors for high-level transgene expression in T lymphocytes. Hum. Gene Ther. *14*, 1155–1168.

48. Cheadle, E.J., Hawkins, R.E., Batha, H., Rothwell, D.G., Ashton, G., and Gilham, D.E. (2009). Eradication of established B-cell lymphoma by CD19-specific murine T cells is dependent on host lymphopenic environment and can be mediated by CD4+ and CD8+ T cells. J. Immunother. *32*, 207–218.

OMTO, Volume *8*

Supplemental Information

CD19 CAR T Cells Expressing IL-12 Eradicate Lymphoma in Fully Lymphoreplete Mice through Induction of Host Immunity

Gray Kueberuwa, Milena Kalaitsidou, Eleanor Cheadle, Robert Edward Hawkins, and David Edward Gilham

Supplementary Figure 1

A)

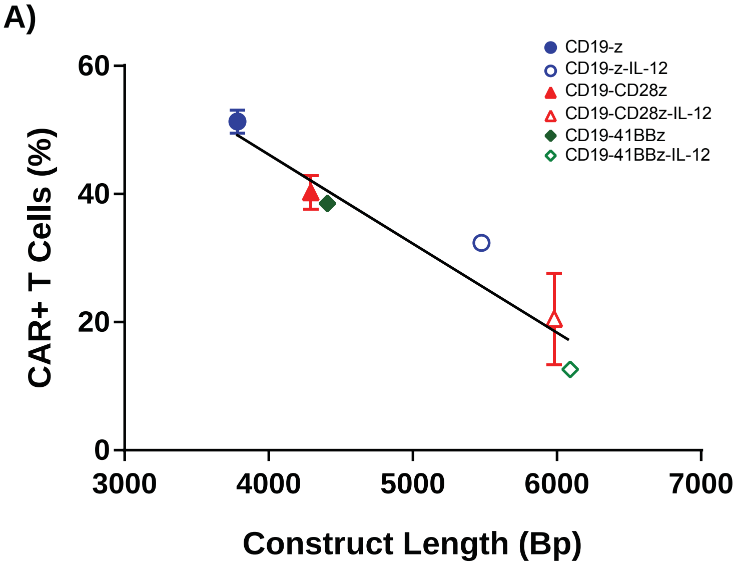

Supplementary Figure 2

A)

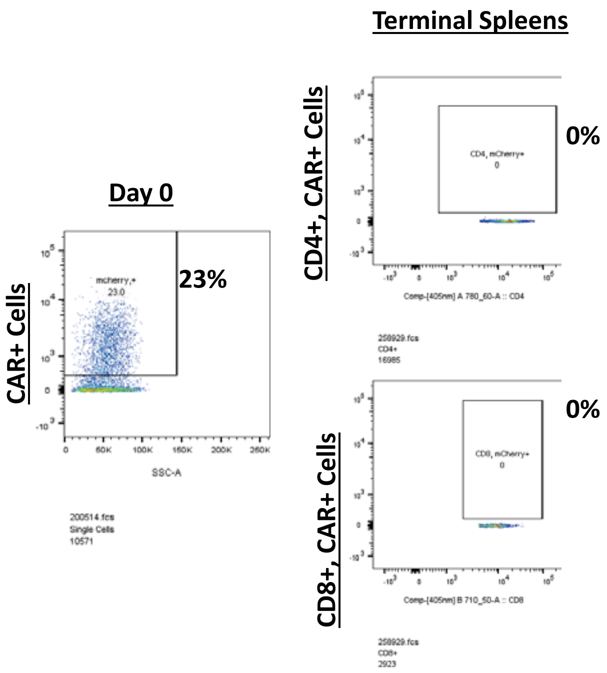

B)

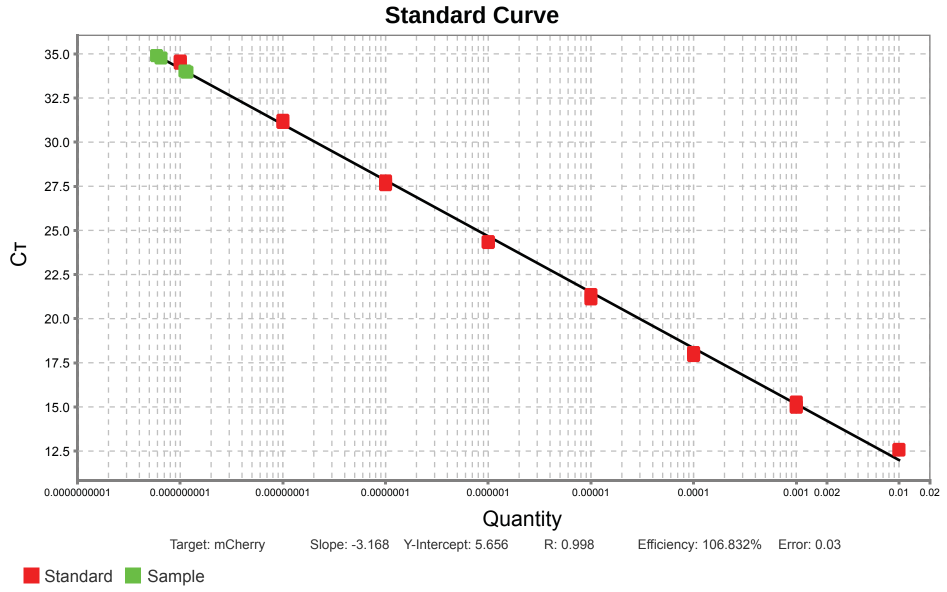

Supplement: Document S2. Article plus Supplemental Information [file mmc2.pdf]
